# Dataset of characterised construction safety risks and related treatments

**DOI:** 10.1016/j.dib.2023.109293

**Published:** 2023-06-04

**Authors:** Carlos A. Osorio-Sandoval, Gordon Crick, William H. Collinge, Karim Farghaly, Mojgan Hadi Mosleh, Patrick Manu, Clara Man Cheung

**Affiliations:** a Department of Civil Engineering, The University of Nottingham, Nottingham NG7 2RD, UK; b Health and Safety Executive, Redgrave Court, Merton Road, Bootle, Merseyside L20 7HS, UK; c Department of Mechanical, Aerospace and Civil Engineering, School of Engineering, The University of Manchester, M13 9PL Manchester, UK; d The Bartlett School of Sustainable Construction, University College London, 1-19 Torrington Place, London, WC1E 7HB, UK; e School of Architecture and Environment, University of the West of England, Bristol, BS16 1QY, UK

**Keywords:** Design for safety, Prevention through design, Risk scenarios, Building information modelling

## Abstract

The Safety Risk Library [1] is a structured database [2] that integrates knowledge drawn from multiple sources to address the problem of information disaggregation in the construction industry. This knowledge base maps construction safety risk scenarios to treatment suggestions that help designers implement the concept of prevention through design. In the context of the Safety Risk Library, risk scenarios are characterised by six data categories based on a formalised ontology [3]. To build the first iteration of the Safety Risk Library, nine different risk scenarios were identified and mapped to relevant risk treatments in focus groups. Subsequently, the Safety Risk Library was pilot tested in six construction projects, and user feedback and input were used to expand the list of risk scenarios and treatment prompts. Additionally, public press releases reporting construction accidents were analysed to identify and characterise risk scenarios, which were then mapped to appropriate treatment suggestions and included in the Safety Risk Library. This dataset can assist construction industry stakeholders in identifying, characterising, communicating and mitigating safety risks in construction projects. It can also be integrated into building information modelling environments to assist designers to implement prevention through design.

**Specifications Table**

**Table:** 

Subject	Safety, Risk, Reliability and Quality
Specific subject area	Safety management in the construction industry
Type of data	Table
How the data were acquired	The first iteration of the Safety Risk Library [1] (Stage 1) was built by identifying nine risk scenarios from the Health and Safety Executive's archive resources (RIDDORs and press releases). Treatments for these scenarios were captured via workshops with an established Steering Committee with industry experts. This first iteration was then deployed within a commercial building information modelling (BIM) platform and pilot tested in six construction projects (Stage 2). Following user feedback, the list of risk scenarios and treatments was further developed. Furthermore, public press releases reporting construction accidents were analysed to identify and characterise risk scenarios. These were mapped to appropriate treatment suggestions and included in the Safety Risk Library via workshops with an established community of practice consisting of members from academia, industry and the HSE (Stage 3).
Data format	Raw Analysed
Description of data collection	Stage 1: Using an ontology [2], HSE press releases and RIDDORs were analysed to identify nine scenarios related to falling from an open edge. Treatments were identified via workshops with an industry Steering Committee and relevant industry sources [4], [5], [6], [7]. Stage 2: The first iteration was deployed in a commercial cloud-based BIM tool and tested in six UK construction projects at design stage. Scenarios and treatments were obtained via user input, and new ones were added following review workshops. Projects ranged in type, including residential, industrial, commercial, and infrastructure projects. Stage 3: Public press releases reporting construction accidents in free text format, providing sufficient information to extract risk scenarios were analysed, characterised and mapped to treatments via workshops with input from an established community of practice consisting of academics, experts and HSE personnel.
Data source location	• Institution: The University of Manchester • City/Town/Region: Manchester • Country: United Kingdom
Data accessibility	Repository name: Mendeley Data [2] Direct URL to data: https://data.mendeley.com/datasets/bmhzshjt9m
Related research article	W.H. Collinge, K. Farghaly, M.H. Mosleh, P. Manu, C.M. Cheung, C.A. Osorio-Sandoval, BIM-based construction safety risk library, Autom. Constr. 141 (2022) 104391. https://doi.org/10.1016/j.autcon.2022.104391.

## Value of the Data

• This database provides access to a foundational library of construction health and safety knowledge to support the implementation of the prevention through design concept. The data is particularly valuable because it was generated through a unique process that draws on both explicit and tacit knowledge in the prevention through design domain. Additionally, the treatments are not specific to a particular design stage, but instead are categorised based on their applicability across various design stages. The database is also structured based on an existing ontology [3]. The library is ready to be used in construction projects and can be further enhanced to include additional information.
• Construction industry stakeholders, particularly designers and health and safety practitioners, can benefit from having access to this database, as it can provide valuable information to improve safety measures on construction sites. Furthermore, researchers in the field of construction health and safety can also benefit from this database, which can be used to conduct further research and develop new strategies for improving safety in the construction industry.
• The database can be used to develop decision support tools for designers and health and safety practitioners in response to the challenges regarding the application of digital technologies for prevention through design, as outlined in [8]. For further insights see [1].

## 1 Objective

This dataset was developed as part of the Construction Risk Library project, a component of the Discovering Safety Programme, a five-year research and development programme led by the HSE (UK) funded by the Lloyd's Register Foundation. The project aims to develop and implement a knowledge base to help designers manage health and safety risk more effectively, contributing to driving down the annual toll of harm and suffering to workers and members of the public who are affected by the activities of the construction industry. This database is related to [1], which presents a tool that implements its first iteration, and adds value to [1] by presenting the full contents of the expanded version of the Safety Risk Library.

## 2 Data Description

**Fig. 1:**
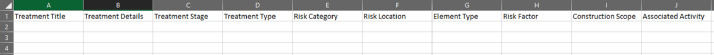
Database structure

The database is structured in ten columns as shown in Fig. 1.

The first four columns describe the suggested treatment as follows:

- Treatment title: Identifies a solution suggested to deal with the related risk scenario.
- Treatment details: If applicable, any additional information about the suggested treatment, such as links to websites illustrating ideas, design best practice, applicable regulations, case studies, etc.
- Treatment stage: Defines the stage at which the suggested treatment can be implemented, which can be preliminary design, detail design, pre-construction, or site work.
- Treatment type: Describes the impact that the treatment can have on the associated risk scenario, which can be eliminate, reduce, control, or inform other stakeholders about the risk.

The remaining six columns characterise the risk scenario as follows:

- Risk category: Identifies the type of risk that could occur in the given scenario.
- Risk location: Identifies the characteristics of the location which can be the reason why a risk arises.
- Element type: Identifies the type of building element associated with the eventuation of the risk.
- Risk factor: Identifies the reason behind the risk eventuation.
- Construction scope: Based on [4], identifies the type of construction work being carried out when the risk could arise. It is divided into five main groups, each of which is subdivided into several categories.
- Associated activity: Identifies the design activity, for example, construction, asset use, maintenance, etc.

## 3 Experimental Design, Materials and Methods

1. *Focus group discussions*

To integrate knowledge from multiple sources, including literature review, the UK Health and Safety Executive (HSE) archive resources, and professional experts, focus group discussion were conducted. The discussions involved two types of focus groups: steering committee workshops and designers’ engagement workshops.

The industry Steering Committee, comprising voluntary members from different construction work groups, such as the BIM4H&S workgroup, was established at the beginning of the project. Members with different professional roles, including construction health and safety, digital information, and design-related roles were invited to participate in two industry committee workshops held in May and November 2019.

Five 90 to 120-minute Designers’ engagement workshops were carried out with participation of 33 experts from three design, engineering, and architecture consultancy firms and two construction and engineering companies. At the beginning of each workshop, different scenarios identified from the HSE archive were introduced and the aim of the workshop was clearly set. The purpose of the workshops was to capture treatment prompts for the identified risk scenarios. Captured information was stored and analysed by the research team.

2. *User input in cloud-based BIM tool implementing the library*

The Safety Risk Library project deployed the database through a novel cloud-based BIM tool that was pilot tested by industry partners. The tool was based on the data structure of the Safety Risk Library and was tested by designers and construction health and safety managers in six construction projects at the early design stage, ranging in type, including, residential, industrial, commercial, and infrastructure projects. During the pilot tests, users would identify risk scenarios in their projects, and input their six characterisation data points (i.e., risk category, risk location, element type, risk factor, construction scope, and associated activity) into the tool's user interface. If the inputted risk scenario was already in the database, the tool would prompt the user to select an appropriate treatment to mitigate the identified risk from the scenario's associated treatments. Otherwise, users would input the treatment that they intended to use in their project to mitigate the identified risk. Risk scenarios and treatments were collected by the research team at the end of the pilot projects and reviewed in workshops with an established community of practice comprising academics, industry practitioners, including tool pilot testers, and HSE staff. In these workshops, risk scenarios were generalised, and further applicable treatments were suggested.

3. *Public press releases analysis*

Press releases related to construction accidents were manually annotated using the below protocol:

- Step 1. Identify the event reported in the press release.
- Step 2. Choose the risk category that best describes the reported event. The chosen category should respond to the question ‘what happened to the subject of the press release?’
- Step 3. Choose the risk factor category that is most strongly linked to the previously selected risk. The chosen category should respond to the question ‘what circumstance eventuated the selected risk?’
- Step 4. Choose the location category that best describes the location of the subject of the press release at the moment of the risk eventuation. The chosen category should respond to the question ‘where was the subject of the press release when the risk eventuated?’ If the question is not clearly responded from the information in the text, and the identified risk could have been eventuated at different locations, leave blank.
- Step 5. Choose the construction scope category that best responds to the question ‘what type of construction work was being carried out at the time of the reported event?’
- Step 6. Choose the activity category that best describes the stage of the life cycle of the asset at the time of the reported event.
- Step 7. Choose the building element category that best describes the building element involved in the reported event. Consider the element that would be most likely present in the asset's design model. If more than one element is identified, follow this priority: element that collapsed or failed (exclude excavation walls collapsing; include temporary structures like scaffolding), element being installed or removed, element being transported or handled.
- Step 8. If applicable, choose more risk categories that could have happened but were not eventuated. Only choose risks related to the location selected in Step 4.
- Step 9. If applicable, choose other risk factors that could have eventuated risks identified in Step 8.

Newly identified risk scenarios were mapped to treatments retrieve from existing construction guidance for designers in the UK [4] or through focus group discussions with the community of practice, as described in stage 1.

## Ethics Statements

The research obtained ethics approval from the University of Manchester Ethics Committee with the protocol number 2018-5118-7959. Subsequently, informed consent was received from the research subjects participating in the steering committee, workshops and piloting the developed tool.

## CRediT authorship contribution statement

**Carlos A. Osorio-Sandoval:** Methodology, Investigation, Data curation, Formal analysis, Writing – original draft. **Gordon Crick:** Conceptualization, Methodology, Investigation, Resources. **William H. Collinge:** Conceptualization, Methodology, Investigation, Writing – review & editing, Supervision, Project administration, Funding acquisition. **Karim Farghaly:** Conceptualization, Methodology, Data curation, Formal analysis, Investigation, Writing – review & editing. **Mojgan Hadi Mosleh:** Conceptualization, Methodology, Writing – review & editing, Funding acquisition. **Patrick Manu:** Conceptualization, Methodology, Writing – review & editing, Funding acquisition. **Clara Man Cheung:** Conceptualization, Methodology, Writing – review & editing, Funding acquisition.

## Declaration of Competing Interest

The authors declare that they have no known competing financial interests or personal relationships that could have appeared to influence the work reported in this paper.

## Data Availability

Safety Risk Library dataset (Original data) (Mendeley Data).
